# Wavelength dependence of ultraviolet light inactivation for SARS-CoV-2 omicron variants

**DOI:** 10.1038/s41598-023-36610-6

**Published:** 2023-06-15

**Authors:** Nahoko Fujimoto, Katsuya Nagaoka, Ichiro Tatsuno, Hisashi Oishi, Makoto Tomita, Tadao Hasegawa, Yasuhito Tanaka, Takahiro Matsumoto

**Affiliations:** 1 Department of Gastroenterology and Hepatology, Faculty of Life Sciences, Kumamoto University, Kumamoto, 860-8556 Japan; 2 Graduate School of Medical Sciences, Nagoya City University, Nagoya, 467-8601 Japan; 3 Department of Physics, Faculty of Science, Shizuoka University, Shizuoka, 422-8529 Japan; 4 Graduate School of Design and Architecture, Nagoya City University, Nagoya, 464-0083 Japan

**Keywords:** Biophysics, Environmental sciences

## Abstract

Ultraviolet (UV) irradiation offers an effective and convenient method for the disinfection of pathogenic microorganisms. However, UV irradiation causes protein and/or DNA damage; therefore, further insight into the performance of different UV wavelengths and their applications is needed to reduce risks to the human body. In this paper, we determined the efficacy of UV inactivation of the SARS-CoV-2 omicron BA.2 and BA.5 variants in a liquid suspension at various UV wavelengths by the 50% tissue culture infection dose (TCID_50_) method and quantitative polymerase chain reaction (qPCR) assay. The inactivation efficacy of 220 nm light, which is considered safe for the human body, was approximately the same as that of health hazardous 260 nm light for both BA.2 and BA.5. Based on the inactivation rate constants determined by the TCID_50_ and qPCR methods versus the UV wavelength, the action spectra were determined, and BA.2 and BA.5 showed almost the same spectra. This result suggests that both variants have the same UV inactivation characteristics.

## Introduction

With the global outbreak of severe acute respiratory syndrome coronavirus 2 (SARS-CoV-2) and the emergence of their new variants, there is a great demand for developing and demonstrating efficient disinfection technologies to protect against various pathogenic viruses and bacteria^1–3^. In this case, vaccines provide effective protection against the infection, the efficacy and supply speed of these vaccines against future emerging SARS-CoV-2 variants are not clear at the present stage^4^. Therefore, it is important to prepare additional strategies to mitigate public health risks during the pre-vaccine development period against emerging pathogens.

Disinfection by ultraviolet (UV) irradiation is attracting special interest to reduce SARS-CoV-2 transmission because UV irradiation offers an effective and convenient method for the inactivation of pathogenic microorganisms, including SARS-CoV-2^5–10^. In particular, the wavelength range from 200 to 235 nm, often referred to as far-UVC, has attracted increasing attention as a novel disinfection wavelength. Far-UVC light shows a strong germicidal effect on pathogenic viruses and bacteria^11–15^ and has been shown to be harmless to mammalian cells due to the strong absorption effect of the stratum corneum layer^16–20^. However, its safety profile in mammalian cells has been much less thoroughly documented, and there are numerous reports suggesting that far-UVC light is not as safe as irradiation far beyond threshold levels^21–26^ since it significantly damages epidermal cells, leading to the formation of erythema and cyclopyrimidine dimers^21–24,26^.

Furthermore, the inactivation dose reported to achieve a certain log-reduction varies widely from approximately 1 to 20 mJ/cm^2^^5,10,27–34^. Such inconsistencies might be caused by varied experimental conditions and setups employed. For example, many light sources, such as UV-LEDs, KrCl-excimer lamps, and metal vapor discharge lamps, have been used to inactivate SARS-CoV-2^5,14,27–34^; however, it is difficult to compare the magnitude of dose and the inactivation efficacy for these different UV wavelength regions due to the differences in both the strains of SARS-CoV-2 and experimental conditions such as the spectrum of the light sources. Therefore, there is a substantial need for systematic experiments with varying UV wavelengths and without variance in other experimental conditions.

In this paper, we describe the inactivation efficacy of the SARS-CoV-2 omicron BA.2 and BA.5 variants in a viral suspension as a function of UV wavelength with 10 nm bandwidth based on the construction of a UV wavelength tunable irradiation source. We employed the standard 50% tissue culture infection dose (TCID_50_) method and a quantitative polymerase chain reaction assay (qPCR) to detect UV damage to the viral genome. We have found a strong correlation between the TCID_50_ and qPCR. Based on the inactivation rate constants determined by the TCID_50_ and qPCR methods versus UV wavelength, the action spectra of both BA.2 and BA.5 were determined, and these two variants showed almost the same spectra. This result suggests that both variants have the same UV inactivation characteristics and that the action spectra of SARS-CoV-2 were quantitatively explained by the absorption spectra of both RNA and protein, where the protein layer shields the RNA from the UV light.

## Materials and methods

### Cells

VeroE6/TMPRSS2 cells (African green monkey kidney-derived cells expressing human TMPRSS2) were obtained from the Japanese Collection of Research Bioresources (JCRB) Cell Bank (#JCRB1819). The cells were cultured in Dulbecco’s modified Eagle’s medium (DMEM, low-glucose, Sigma–Aldrich, #D6046) supplemented with 10% fetal bovine serum (Gibco, #10270-106), penicillin/streptomycin (Sigma–Aldrich, #P0781), and 1 mg/mL G418 (Wako, #070-06803) at 37 °C with 5% CO_2_. The concentration of cells was approximately 1.4 × 10^5^ cells/cm^2^.

### Virus preparation, stocks and infectivity assays

Two types of SARS-CoV-2 variants, omicron BA.2 (hCoV-19/Japan/TKYS02037/2022) and omicron BA.5 (hCoV-19/Japan/TKYS14631/2022), were obtained from Tokyo Metropolitan Institute of Public Health. These viruses were propagated in VeroE6/TMPRSS2 cells cultured in medium A (DMEM containing penicillin/streptomycin and 1 mg/mL G418) for infection and incubated for 3 days at 37 °C with 5% CO_2_. After infection, the virus-containing supernatant was collected and the cellular debris was removed by centrifugation at 3000 rpm (= 1700 g) for 5 min. The virus stocks were then aliquoted and stored at − 80 °C until use. We measured viral infectivity with the standard TCID_50_ method to determine the viral titer of the collected viral samples. TCID_50_/mL values were calculated 4 days after the infection using the Behrens–Karber method^35^. The viral titer of BA.2 was 4.9 × 10^5^ TCID_50_/mL and that of BA.5 was 2.1 × 10^5^ TCID_50_/mL.

### Quantitative reverse transcription polymerase chain reaction (RT‒qPCR)

SARS-CoV-2 RNA was extracted from the collected viral samples of each well using TRIzol Reagent following the manufacturer’s protocol. RT‒qPCR for SARS-CoV-2 was performed using a PrimeScript RT Reagent Kit with gDNA Eraser (Takara Bio Inc., #RR047A) according to the manufacturer’s protocol. qPCR was performed using TB Green Premix Ex Taq (Takara Bio Inc., #RR420A) according to the manufacturer’s protocol. The following primers were used: qCoV2 forward, 5´-GCCTCTTCTCGTTCCTCATCAC-3´; qCoV2 reverse, 5´-AGCAGCATCACCGCCATTG-3´; Glyceraldehyde-3-phosphate dehydrogenase (GAPDH) forward, 5´-ACACCCACTCCTCCACCTTT-3´; and GAPDH reverse, 5´-TAGCCAAATTCGTTGTCATACC-3´. Thermal cycling was carried out as follows: initial denaturation at 95 °C for 30 s, 40 cycles of denaturation at 95 °C for 5 s, and a final annealing/extension at 60 °C for 30 s. For BA.2, the value of the threshold cycle (C_t_) without UV irradiation was C_t_ = 13 and that with UV irradiation (260 nm, 18 mJ/cm^2^) was C_t_ = 16. For BA.5, C_t_ = 13 without UV irradiation and C_t_ = 17 with UV irradiation (260 nm, 18 mJ/cm^2^). In both cases, we used GAPDH as a reference gene and its C_t_ value was 27. As a dye for staining DNA, we used SYBR^@^ FAM for the fluorescence detection. We set the fluorescent intensity as level 10 (Thermal Cycler Dice Real Time System Software, Thermo Fisher Scientific Inc. Massachusetts, USA) to determine all C_t_ values. All experiments with SARS-CoV-2 were performed in a biosafety level 3 (BSL3) containment facility at Kumamoto University.

### Plating and counting method for inactivated virus

We applied from 200 to 260 nm-UV irradiation to inactivate virus suspensions. For each wavelength, we varied dose from 0 to 18 mJ/cm^2^. A total of 600 μL of viral suspension (200 μL of virus stock mixed with 400 μL of PBS) was irradiated for each wavelength and dose. VeroE6/TMPRSS2 cells were plated in both 96-well plates for TCID_50_ assays and 24-well plates for qPCR one day prior to infection. Just before the infection experiments, the medium on VeroE6/TMPRSS2 cells was aspirated, and 50 μL of the medium A was added to each well. We used the TCID_50_ method to determine viral infectivity. Inactivated virus or control virus suspensions were plated into the first column, and then the threefold-diluted suspensions were successively plated into the adjacent columns. This dilution plating was performed for all 96 wells. The plated 96 wells were incubated for one hour at 37 °C. After incubation, the viral supernatants were aspirated, and 100 μL of medium B (DMEM supplemented with 10% FBS, penicillin/streptomycin, and 1 mg/mL G418) for culture was added to each well. The plate was incubated for four days at 37 °C in a 5% CO_2_. Cytopathic effects (CPEs) were scored under a bright field microscope (10 ×) as cytoplasm vacuolization, cell rounding and sloughing. TCID_50_/mL values were calculated by the Behrens–Karber method^35^. Viral genome integrity was analyzed using reverse-transcription quantitative-polymerase chain reaction (RT‒qPCR). We used the same inactivated virus suspensions as those used in the TCID_50_ assay without dilution. A 100 μL viral suspension was plated on VeroE6/TMPRSS2 cells in a 24-well multiwell dish. This plate was incubated for one hour at 37 °C in a 5% CO_2_. After incubation, the viral supernatants were aspirated, and then 500 μL of the medium B was added to each well. This plate was incubated for one day at 37 °C in a 5% CO_2_. SARS-CoV-2 RNA was extracted from the collected viral samples of each plate using TRIzol Reagent (Thermo Fisher, #15,596,018). All experimental results are reported as the means across 3 replicates.

### Wavelength-tunable UV light source

**Figure 1:**
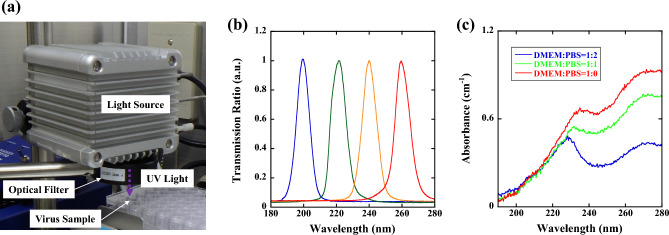
(**a**) Optical setup of the wavelength-tunable UV inactivation system, (**b**) transmission spectrum, and (**c**) absorbance spectra of DMEM diluted with PBS. A laser-driven light source, which emits radiation of 170–2100 nm, was used as a broadband emission source, and the emission was selected by using a UV bandpass filter from 200 to 260 nm with a 10 nm bandwidth. We used a DMEM:PBS = 1:2 solution (blue line) because the absorbance between 200 and 260 nm is approximately the same magnitude.

Figure 1a shows the wavelength-tunable UV light source used to compare the efficacy of the far-UV light region (200–230 nm) and the deep UV (DUV) light region (230–260 nm). A Laser-Driven Light Source (LDLS EQ-99X, Energetiq Technology, Inc. Wilmington, USA), which emits radiation of 170–2100 nm, was used as a broadband emission source. The emission was selected by using a UV bandpass filter from 200 to 260 nm (200 nm, 220 nm, 240 nm, and 260 nm) with a 10 nm bandwidth (Edmond Optics Japan, Tokyo, Japan). The spectrum of UV radiation that the virus was exposed to, which is shown in Fig. 1b, was measured by a spectrometer through an optical fiber. The viral suspension (600 μL) was added to a single well (10 mm in diameter) of a microplate (48 wells), and during UV irradiation, the viral suspension was agitated by a microplate shaker (TM-1FN, AS ONE Corp. Osaka, Japan). The irradiance of UV radiation that the virus was exposed to was measured by setting a UV-extended Si photodiode with an aperture of 10 mm (S120VC, Thorlabs Inc. New Jersey, USA) at the surface of the viral suspension.

### Absorbance correction of viral suspensions

Generally, the medium A is used to maintain viral viability and infectivity, and this medium contains proteins and amino acids which strongly absorb UV light^36–38^. To extract the viral particles from the medium A, an ultracentrifugation followed by buffer exchange can be used. However, various problems such as the shed of the S protein during the ultracentrifugation are pointed out^39–41^. Therefore, to measure correct viability and infectivity versus UV irradiation, the absorbance of the viral solution was adjusted by PBS dilution^42^. The absorbance spectra of DMEM diluted with PBS were measured using a UV–visible spectrometer through an optical fiber (BIM-6002A, Brolight Technology Corporation, Hangzhou, China). Here, all the absorbance spectra were calibrated by a spectral calibrated 150 W-xenon standard light source with an emission wavelength from 185 to 2000 nm (L7810-03, Hamamatsu Photonics Corporation, Hamamatsu, Japan), and a fused silica cell with an optical path length of 1 cm (T-3-ES-10, Tosoh Quartz Corporation, Tokyo, Japan) was used for the absorbance measurements. As shown in Fig. 1c, DMEM exhibited absorption peaks at approximately 230 nm and 280 nm, which were due to proteins or amino acids in DMEM. The absorbance by PBS (not shown), which contains NaCl, KCl, and sodium phosphate, was much lower than 0.1 cm^−1^ at all wavelengths (200–300 nm). To avoid the influence of absorption by DMEM components, the viral suspension could be diluted with PBS. However, in this case, the virus titer would also decrease. Therefore, we used a DMEM:PBS = 1:2 solution (blue line), for which the absorbance at 200 nm, 220 nm, 240 nm, and 260 nm did not show significantly different magnitudes (α_200 nm_ = 0.12 cm^−1^, α_220 nm_ = 0.32 cm^−1^, α_240 nm_ = 0.30 cm^−1^, and α_260 nm_ = 0.35 cm^−1^). The viral suspension used here was 0.6 cm in height (L). The irradiance that the virus was exposed to differed up to 30% between the top and bottom layers; e.g. 30 µW/cm^2^ for the top layer and 20 µW/cm^2^ for the bottom layer at the wavelength of 260 nm. Therefore, to determine the effective irradiance (I_e_) correctly, we subtracted the reflection loss (R) at the air/suspension interface and averaged the absorption effect in the height direction as

1 $${I}_{e}=\left(1-R\right)\frac{{I}_{0}}{L}{\int }_{0}^{L}\mathrm{exp}\left(-\alpha x\right)dx=\frac{{I}_{0}}{\alpha L}(1-R)\left[1-\mathrm{exp}\left(-\alpha L\right)\right] ,$$

where I_0_ is the irradiance measured at the top layer. The reflection loss was determined by using the Fresnel equation^43^, and R is approximately 0.02 to 0.03, as we assume that the refractive index of the suspension has a value similar to that of water^44,45^. Based on the irradiance determined by Eq. (1), the dose was varied from 0 to 18 mJ/cm^2^ (0 mJ/cm^2^, 3 mJ/cm^2^, 6 mJ/cm^2^, 9 mJ/cm^2^ and 18 mJ/cm^2^) by changing the UV irradiation duration.

## Ethical approval statement

All protocols were reviewed and approved by the Ethics Committee for Faculty of Life Science, Kumamoto University (approval number 49).

## Results

### Dose response of SARS-CoV-2 at various wavelengths measured by infectivity and RNA amplification

**Figure 2:**
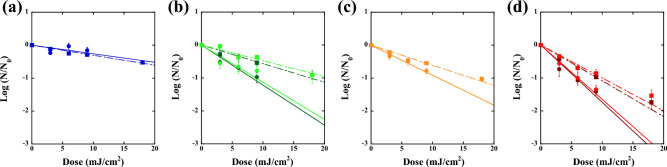
Inactivation of SARS-CoV-2 BA.2 and BA.5 using the wavelength-tunable UV light source. Inactivation at (**a**) 200 nm (BA.2; blue), (**b**) 220 nm (BA.2; dark green, BA.5; light green), (**c**) 240 nm (BA.2; orange), and (**d**) 260 nm (BA.2; dark red, BA.5; light red) as a function of UV dose. Solid circles show the viral infectivity obtained by TCID_50_ assay, and solid squares show the reduction in RNA amplification determined by qPCR, where the relative ratio to those of unexposed controls was used. Inactivation rate constants at each wavelength were determined by linear regression lines (solid line: TCID_50_, broken line: qPCR). These linear inactivation rate constants for each wavelength are summarized in Table 1.

Inactivation of SARS-CoV-2 omicron BA.2 and BA.5 using the wavelength tunable UV light source are presented in Fig. 2(a; 200 nm), (b; 220 nm), (c; 240 nm), and (d; 260 nm) as a function of UV dose. According to comparison of Fig. 2b,d, omicron BA.2 and BA.5 show approximately the same reduction in viral infectivity (solid circles) and in RNA amplification (solid squares) for both 220 nm (BA.2: dark green, BA.5: light green) and 260 nm (BA.2: dark red, BA.5: light red) UV irradiation. This result indicates that the BA.2 and BA.5 variants have almost the same UV irradiation inactivation properties. The fact that the inactivation rates obtained with 220 nm light show approximately the same value as that obtained with 260 nm light highlights the significance of disinfection by far-UVC light because far-UVC light is attracting special attention as a safe germicidal light for the human body^16–20^.

Figure 2 shows a correlation between the reduction in viral infectivity (solid circles) and the reduction in RNA amplification (solid squares) for these wavelengths of UV irradiation. The highest inactivation rate constant (Γ, cm^2^/mJ) was obtained at 260 nm; for cell culture infectivity, the rate of BA.2 was 0.40 (*p* < 0.05), and that of BA.5 was 0.38 (p < 0.05); for the qPCR assay analyzing 111 bp fragments, the rate of BA.2 was 0.25 (p < 0.05), and the rate of BA.5 was 0.23 (p < 0.05). These linear inactivation rate constants (cm^2^/mJ) for each wavelength are summarized in Table 1. As shown in Fig. 2 and Table 1, the obtained rates are different between TCID_50_ and qPCR. However, there is a correlation between these rate constants. Both the difference and the correlation between TCID_50_- and qPCR-rates are considered to originate from the fact that the TCID_50_ measures the number of infectious virus, while the qPCR measures both non-infectious and infectious viruses.

**Table 1:** Inactivation rate constants (cm^2^/mJ) for 200 nm, 220 nm, 240 nm, and 260 nm determined by TCID_50_ and qPCR experiments.

	200 nm	220 nm	240 nm	260 nm
TCID_50_	0.06	0.28, 0.26 (BA.5)	0.21	0.40, 0.38 (BA.5)
qPCR	0.07	0.13, 0.11 (BA.5)	0.14	0.25, 0.23 (BA.5)

### Determination of the spectral sensitivities of SARS-CoV-2

**Figure 3:**
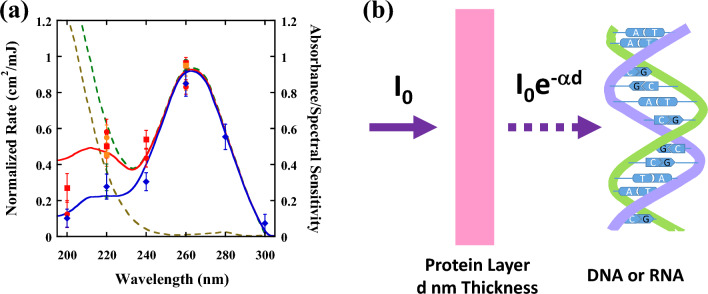
(**a**) Spectral sensitivity (action spectra) of SARS-CoV-2 BA.2 and BA.5 inactivation (BA.2: red circles, BA.5: orange circles) and genome damage (BA.2: red squares, BA.5: orange squares), obtained by calculating the inactivation rate constants relative to their peak values at 260 nm. Both the spectral sensitivities obtained by TCID_50_ assay and by qPCR coincide with each other after multiplying the inactivation rate constants obtained by qPCR by 1.6. As a comparison, the spectral sensitivity of *E. coli* determined by CFU experiments (blue rhombus) is also shown. The solid blue line and solid red line are theoretically fitted action spectra for SARS-CoV-2 (red line) and *E. coli* (blue line) determined by weighting the absorption coefficient of the protein layer (broken brown line) to that of DNA or RNA (broken green line), where the action spectra for SARS-CoV-2 is fitted by α_SARS_ (λ) = α_DNA_ (λ)–1.1 × α_PROTEIN_ (λ) (red line) and α_E. Coli_ (λ) = α_DNA_ (λ)–0.85 × α_PROTEIN_ (λ) (blue line), respectively. (**b**) UV irradiance (mW/cm^2^) shield model for a protein layer to explain the difference in the spectral sensitivity between SARS-CoV-2 and *E. coli* below 240 nm.

Figure 3a shows the spectral sensitivity (action spectra) of SARS-CoV-2 omicron BA.2 inactivation (red circles) and genome damage (red squares) and of omicron BA.5 inactivation (orange circles) and genome damage (orange squares) determined by calculating the inactivation rate constants relative to their peak values at 260 nm. Both the spectral sensitivities obtained by TCID_50_ assays and those obtained by qPCR assays coincide when multiplying the inactivation rate constants obtained by qPCR by 1.6, which shows the correlation between these methods. Notably, the obtained spectral sensitivity is almost identical to that obtained by Schuit et al.^7^.

As a comparison, the spectral sensitivity of *Escherichia coli* (*E. coli*) determined by a colony-forming unit (CFU) experiment (blue rhombus) is also shown in this figure^46^. The figure highlights approximately the same value for SARS-CoV-2 BA.2 and BA.5 inactivation, SARS-CoV-2 BA.2 and BA.5 genome damage, and *E. coli* inactivation, namely, above 240 nm. These values are aligned with the absorbance spectrum of RNA, which is shown by the green broken line^47,48^. However, the inactivation rate constants as well as the genome damage show significant differences between SARS-CoV-2 variants and *E. coli* below 240 nm.

## Discussion

If we consider that both RNA and protein absorbance play a role in inactivation, this difference below the 240 nm region can be quantitatively understood by considering the thickness of the protein layer covering DNA or RNA, as shown in Fig. 3b. That is, *E. coli* DNA is covered by a thick protein layer, whereas SARS-CoV-2 RNA is covered by a thin protein layer. This protein layer strongly absorbs UV light below 240 nm (shield effect); thus, the UV irradiance (mW/cm^2^) of *E. coli* DNA is significantly reduced compared to that to SARS-CoV-2 RNA. The solid blue line and solid red line shown in Fig. 3a are theoretically fitted action spectra for SARS-CoV-2 (red line) and *E. coli* (blue line) determined by weighting the absorption coefficient of the protein layer (broken brown line) to that of DNA or RNA (broken green line)^47,48^, where the action spectra for SARS-CoV-2 is fitted by α_SARS_ (λ) = α_DNA_ (λ)–1.1 × α_PROTEIN_ (λ) and α_*E. coli*_ (λ) = α_DNA_ (λ)–0.85 × α_PROTEIN_ (λ), respectively. Notably, the above theoretical analysis is based on the fact that the excitation of peptide bonds plays a minor role in both RNA modification and bacterial inactivation because protein consists of a much larger number of molecules than DNA and protein can be repaired using genetic information when necessary^49–51^.

As shown in Table 1, we determined the linear inactivation rate constants (cm^2^/mJ) of BA.2 and BA.5 for each UV wavelength. These values were obtained using a viral suspension and were significantly different from the values obtained for coronavirus in aerosols^12^. For example, there is a large difference in the inactivation rate constant at 220 nm obtained here (suspension: 0.28 cm^2^/mJ) and that reported by Buonanno et al. (aerosol: 4–6 cm^2^/mJ)^12^. It is likely that some physical and/or biochemical mechanisms are responsible for this large difference. We note here that this large difference between aerosol and liquid suspensions is widely recognized for many viruses, such as SARS-CoV^10,52^, murine hepatitis virus (MHV) coronavirus^53^, adenovirus serotype 2 (VR-846)^53^, influenza virus H1N1^13,54^, and bacteriophage MS2^53^. This comparison shows the definite enhancement of efficacy in aerosols compared to that in liquid suspensions regardless of the size of the virus (90–100 nm or 30–40 nm), the type of nucleic acid (DNA or RNA), and the viral structure (naked or enveloped). The difference is quantitatively understood based on the optical Mie scattering theory^55,56^. Our calculation shows that the inactivation rate constant in the aerosol state is enhanced by a factor of 10 compared to that in the liquid suspension. The quantitative evaluation of the enhancement factor as a function of the droplet size is provided in Supplemental information (Fig. S1). The Mie scattering effect is therefore a possible candidate to explain this significant enhancement of the UV irradiance inside an aerosol droplet.

The inactivation rate constants obtained at 220 nm is smaller than those obtained at 260 nm. However, if we consider the threshold level that can be irradiated to the human body^57,58^, far-UVC radiation (220 nm) can be effective compared to Deep-UVC radiation (260 nm). For example, the total amount of UV radiation that can be irradiated per day is 25 mJ/cm^2^ for 220 nm, and 3 mJ/cm^2^ for 260 nm^57,58^. Multiplying these values by the rate constants obtained here yields a 3-log inactivation efficacy at 220 nm, whereas only 30% inactivation efficacy can be obtained by 260 nm irradiation. Therefore, considering the safety level to the human body, far-UVC can efficiently inactivate SARS-CoV-2 compared to generally used UVC wavelength region (250–270 nm).

## Conclusions

To our knowledge, this is the first investigation of the effect of the UV susceptibility of SARS-CoV-2 omicron BA.2 and BA.5. We determined the inactivation rate constant by TCID_50_ and qPCR methods as a function of UV irradiation wavelength. The spectral sensitivity of SARS-CoV-2 omicron variants was derived from these inactivation rate constants. Difference in the inactivation rate constants obtained by TCID_50_ and qPCR is an issue to be resolved. The fact that the inactivation efficacy of 220 nm light is approximately the same as that of 260 nm light shows a promising aspect that far-UVC light can be used to prevent airborne virus transmission in a simple and safe manner.

## Supplementary Information

Supplementary Information.

## Data Availability

The datasets used and/or analyzed during the current study are available from the corresponding author on reasonable request.
