# Supplementary material for: Wavelength dependence of ultraviolet light inactivation for SARS-CoV-2 omicron variants
Source: Sci Rep. 2023 Jun 15;13:9706. doi: 10.1038/s41598-023-36610-6 (PMC10272214; doi:10.1038/s41598-023-36610-6)
Supplement: Supplementary file 1 — Supplementary Information. [file 41598_2023_36610_MOESM1_ESM.doc]

**Supplemental Information**

-------------------------------------------------------------------------------------------------------------------------------------

**Wavelength dependence of ultraviolet light inactivation for SARS-CoV-2 omicron variants**

Nahoko Fujimoto^a^, Katsuya Nagaoka^a^, Ichiro Tatsuno^b^, Hisashi Oishi^b^, Makoto Tomita^c^,

Tadao Hasegawa^b^, Yasuhito Tanaka^a^ & Takahiro Matsumoto^a,b,d^,*

*^a^Department of Gastroenterology and Hepatology, Faculty of Life Sciences, Kumamoto University, Kumamoto 860-8556, Japan.*

*^b^Graduate School of Medical Sciences, Nagoya City University, Nagoya 467-8601, Japan.*

*^c^Department of Physics, Faculty of Science, Shizuoka University, Shizuoka 422-8529, Japan.*

*^d^Graduate School of Design and Architecture, Nagoya City University, Nagoya 464-0083, Japan.*

*Author to whom correspondence should be addressed: Graduate School of Design and Architecture, Nagoya City University, Nagoya 464-0083, Japan.

Email address: matsumoto@sda.nagoya-cu.ac.jp (T. Matsumoto)

**Quantitative evaluation of the irradiance enhancement in a droplet using Mie scattering theory**

As shown in Table 1, we determined the linear inactivation rate constants (cm^2^/mJ) of BA.2 and BA.5 for each UV wavelength. These values were obtained using a viral suspension and were significantly different from the values obtained for coronavirus in aerosols [1]. For example, there is a large difference in the inactivation rate constant at 220 nm obtained here (suspension: 0.28 cm^2^/mJ) and that reported by Buonanno et al. (aerosol: 4-6 cm^2^/mJ) [1]. Although there are differences in the strains of coronavirus, such as αHCoV-229E and βHCoV-OC43 [1] and SARS-CoV-2 omicron BA.2 and BA.5, we consider that there is not a significant difference in RNA structure; it is likely that some physical and/or biochemical mechanisms are responsible for this large difference. Furthermore, this large difference between aerosol and liquid suspensions is widely recognized for many viruses, such as SARS-CoV [2,3], murine hepatitis virus (MHV) coronavirus [4], adenovirus serotype 2 (VR-846) [4], influenza virus H1N1 [5,6], and bacteriophage MS2 [4]. A comparison of the inactivation rate constants and the ratio between those observed for aerosols and liquid suspensions for SARS-CoV, MHV-CoV, H1N1, adenovirus, and MS2 at 220 nm and 254 nm is shown in Table S1. This comparison shows the definite enhancement of efficacy in aerosols compared to that in liquid suspensions regardless of the size of the virus (90-100 nm or 30-40 nm), the type of nucleic acid (DNA or RNA), and the viral structure (naked or enveloped).

Here, we consider the enhancement based on Mie scattering theory [7,8]. The volume-averaged irradiance, *I* (*a*) (mW/cm^2^), inside the aerosol sphere with radius *a* can be expressed as

where ε_1_ and μ_1_ are the permittivity and permeability at the considered wavelength (λ), respectively, and the electric (***E***) and magnetic (***H***) vectors inside an aerosol sphere are integrated in a spherical coordinate system (θ, φ, r). The inset of Figure S1 shows a graphical image of the electromagnetic field inside the sphere (*a*=200 nm). It is found that the magnitude of the irradiance inside the sphere depends on the radial position and that the irradiance at the outer part of the sphere (*r*>0.8*a*) is significantly enhanced by one order of magnitude compared to that of incident irradiance. When we average the electromagnetic field inside the whole sphere region by using equation (S1), this averaged irradiance also becomes enhanced. The solid line in Figure S1 shows the averaged irradiance normalized by the irradiance in the liquid suspension (*I* (*a*)/*I*_0_), where the horizontal axis is defined as the size parameter *x*=2π*a*/λ. We are unsure of the size of the aerosol, but when we assume the aerosol size to be *a*=200 nm [1,9] and the incident field wavelength to be 222 nm, the size parameter becomes *x*=5.66. In this case, the averaged irradiance in an aerosol becomes enhanced by a factor exceeding 2 compared to that of the irradiance in a suspension. We have no information about the virus position inside the aerosol; however, based on Mie scattering theory, the inactivation rate constant in an aerosol is enhanced by a factor from 2 to 10 compared to that in liquid suspension. The Mie scattering effect is therefore a possible candidate to explain this significant enhancement of the UV irradiance inside an aerosol droplet. A much larger enhancement is possible in a larger aerosol droplet; thus, the combination of aerosol droplets (water mist particles) and harmless far-UVC light could significantly reduce the disinfection dose. This combination can be applied for not only SARS-CoV-2 but also various future emerging airborne pathogenic viruses. Thus, this combination seems to be a promising approach toward a novel and efficient air disinfection method to prevent airborne infections in places where many people gather without overexposure to UV light.

**References**

[1] Buonanno, M., Welch, D., Shuryak, I. & Brenner, D.J. Far-UVC light (222 nm) efficiently and safely inactivates airborne human coronaviruses. *Sci. Rep.* **10**, 10285 (2020).

[2] Heßling, M., Hönes, K., Vatter, P. & Lingenfelder, C. Ultraviolet irradiation doses for coronavirus inactivation – review and analysis of coronavirus photoinactivation studies. *GMS Hyg. Infect. Control* **15**, Doc08 (2020).

[3] Ueki, H., Ito, M., Furusawa, Y., Yamayoshi, S., Inoue, S. & Kawaoka, Y. A 265-Nanometer High-Power Deep-UV Light-Emitting Diode Rapidly Inactivates SARS-CoV-2 Aerosols. *mSphere* **7**, e00941-21 (2022).

[4] Walker, C.M. & Ko, G. Effect of Ultraviolet Germicidal Irradiation on Viral Aerosols. *Environ. Sci. Technol.* **41**, 5460–5465 (2007).

[5] Narita, K., Asano, K., Naito, K., Ohashi, H., Sasaki, M., Morimoto, Y., Igarashi, T. & Nakane, A. Ultraviolet C light with wavelength of 222 nm inactivates a wide spectrum of microbial pathogens. *J. Hosp. Infect.* **105**, 459-467 (2020).

[6] McDevitt, J.J., Rudnick, S.N. & Radonovich, L.J. Aerosol susceptibility of influenza virus to UV-C light. *Appl. Environ. Microbiol.* **78**, 1666-1669 (2012).

[7] Bohren, C.F & Donald R. Huffman, D.R. *Absorption and Scattering of Light by Small Particles.* Wiley, New York, 1983.

[8] Bott, A. & Zdunkowski, W. Electromagnetic energy within dielectric spheres. *J. Opt. Soc. Am. A* **4**, 1361-1365 (1987).

[9] Welch, D., Buonanno, M., Grilj, V., Shuryak, I., Crickmore, C., Bigelow, A. W., Randers-Pehrson, G., Johnson, G. W. & Brenner, D. J. Far-UVC light: A new tool to control the spread of airborne-mediated microbial diseases. *Sci. Rep.* **8**, 2752 (2018).

**Figure and Table**

**
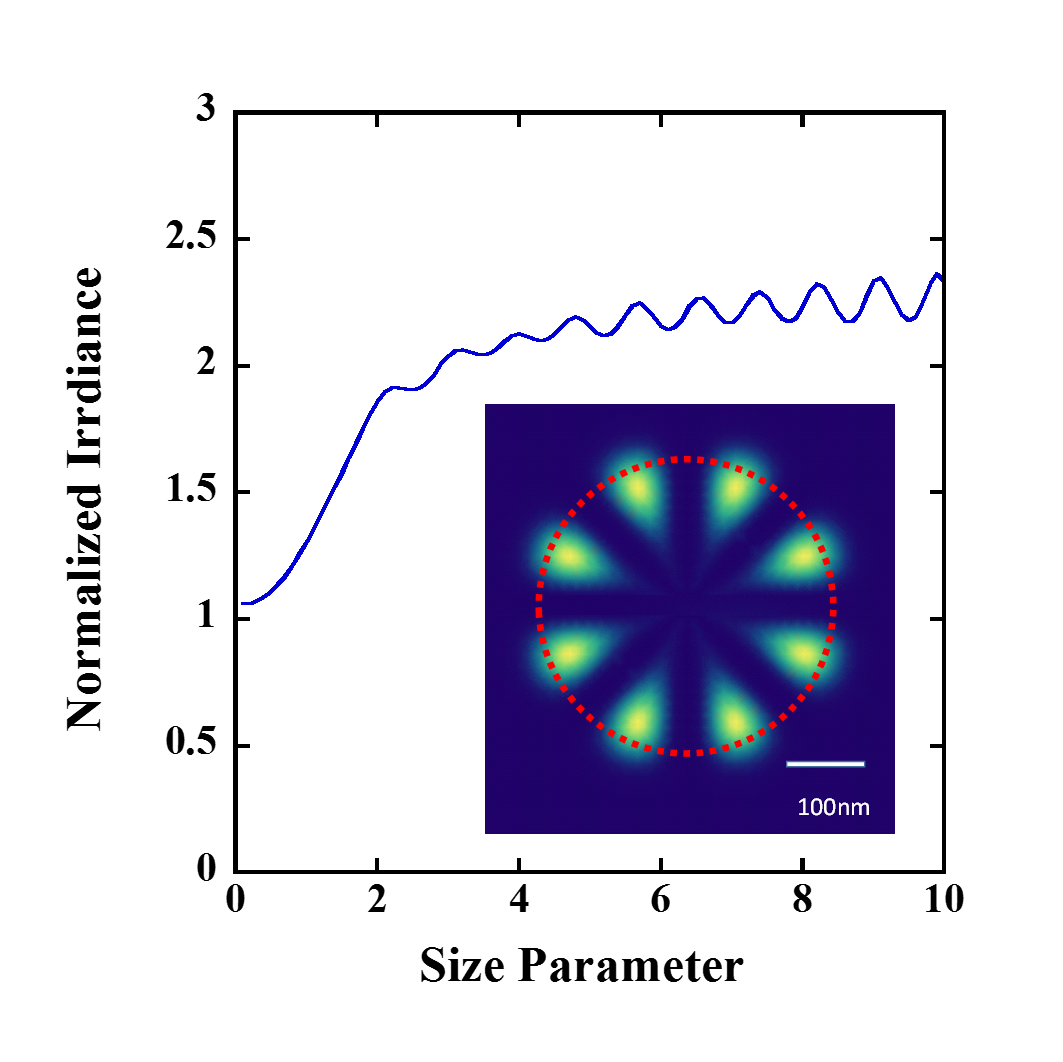
**

**Figure S1**. Normalized irradiance versus size parameter (blue line), x=2πa/λ, calculated based on Mie scattering theory to explain the optical field enhancement in an aerosol droplet. The inset shows an example of the field pattern (mode number: 4) inside an aerosol droplet with r=200 nm.

**Table S1**. Comparison of the inactivation rate constant (cm^2^/mJ) and the ratio between aerosols and liquid suspensions for SARS-CoV, MHV-CoV, H1N1, adenovirus, and MS2 at 220-222 nm and 254 nm.

| **Virus** | **UV Wavelength** | **κ (cm^2^/mJ): Liquid** | **κ (cm^2^/mJ): Aerosols** | **Enhancement Ratio** |
| --- | --- | --- | --- | --- |
| SARS-CoV | 220 nm-222 nm | 0.26-0.28 [This study] | 4-6 [1] | 14-23 |
| MHV-CoV | 254 nm | 0.044 [4] | 3.8 [4] | 86 |
| H1N1 | 222 nm | 0.6 [5] | 1.5-2.1 [9] | 2.5-3.5 |
| H1N1 | 254 nm | 0.6 [5] | 2.2-2.9 [6] | 3.7-4.8 |
| Adenovirus | 254 nm | 0.018 [4] | 0.39 [4] | 21.7 |
| MS2 | 254 nm | 0.055 [4] | 0.38 [4] | 6.9 |
